# Pharmaceutical Hydrates Analysis—Overview of Methods and Recent Advances

**DOI:** 10.3390/pharmaceutics12100959

**Published:** 2020-10-11

**Authors:** Ewa Jurczak, Anna Helena Mazurek, Łukasz Szeleszczuk, Dariusz Maciej Pisklak, Monika Zielińska-Pisklak

**Affiliations:** 1 Department of Physical Chemistry, Chair and Department of Physical Pharmacy and Bioanalysis, Faculty of Pharmacy, Medical University of Warsaw, Banacha 1 str., 02-093 Warsaw, Poland; ejurczak@wum.edu.pl (E.J.); anna.mazurek@wum.edu.pl (A.H.M.); dpisklak@wum.edu.pl (D.M.P.); 2 Department of Biomaterials Chemistry, Faculty of Pharmacy, Medical University of Warsaw, Banacha 1 str., 02-093 Warsaw, Poland; mpisklak@wum.edu.pl

**Keywords:** hydrates, anhydrous, hydration, dehydration

## Abstract

This review discusses a set of instrumental and computational methods that are used to characterize hydrated forms of APIs (active pharmaceutical ingredients). The focus has been put on highlighting advantages as well as on presenting some limitations of the selected analytical approaches. This has been performed in order to facilitate the choice of an appropriate method depending on the type of the structural feature that is to be analyzed, that is, degree of hydration, crystal structure and dynamics, and (de)hydration kinetics. The presented techniques include X-ray diffraction (single crystal X-ray diffraction (SCXRD), powder X-ray diffraction (PXRD)), spectroscopic (solid state nuclear magnetic resonance spectroscopy (ssNMR), Fourier-transformed infrared spectroscopy (FT-IR), Raman spectroscopy), thermal (differential scanning calorimetry (DSC), thermogravimetric analysis (TGA)), gravimetric (dynamic vapour sorption (DVS)), and computational (molecular mechanics (MM), Quantum Mechanics (QM), molecular dynamics (MD)) methods. Further, the successful applications of the presented methods in the studies of hydrated APIs as well as studies on the excipients’ influence on these processes have been described in many examples.

## 1. Introduction

Polymorphism is a phenomenon defined as the possibility of one chemical substance to exist in several different crystallographic forms [1] depending on the temperature, pressure, and humidity as well as solvents applied during the crystallization process. Polymorphic forms of active pharmaceutical ingredients (APIs) may differ in certain important properties, such as solubility in water, dissolution rate, melting point, stability, tabletability, and others, which consequently can have an influence on the drug stability and bioavailability [1]. Different group of structures, although in some respects similar to polymorphs, are hydrates. They form a subtype of solid solvates in which water molecules are incorporated into the crystal lattice of a compound [2]. In comparison with related anhydrates, they exhibit a different structure, as it may be altered by the complex H-bonding network [3]. As a consequence, they may also have different physical and chemical properties than their anhydrous counterparts. These parallels to polymorphs are the reasons hydrates were previously called pseudo-polymorphs [4,5]. This naming can be found in old manuscripts, however, nowadays it is no more accepted as the correct one in relation to hydrates. The topic becomes even more complicated when a particular hydrate in itself has different polymorphic forms, for example, nitrofurantoin monohydrate or niclosamide monohydrate [6]. In such cases, the stoichiometry of the system, the number of both host molecules and water molecules, is preserved and only changes in the arrangement of the constituents in the crystal lattice, and alterations in unit cell parameters occur as well.

Hydrates are of particular interest among solid APIs solvates for several reasons. First, the unique character of the water molecule—its relatively small size and the possibility to form the interactions as both a donor and acceptor of H-bonding, sometimes simultaneously, make it an important “building material” in the field of crystal engineering. Further, from the pharmaceutical point of view, it is a non-toxic substance, in contrast to most of the other organic solvents. Finally, owing to the present of the moisture in the air, spontaneous hydration may occur at any stage of drug production or storage, leading to hydrate formation.

**Figure 1:**
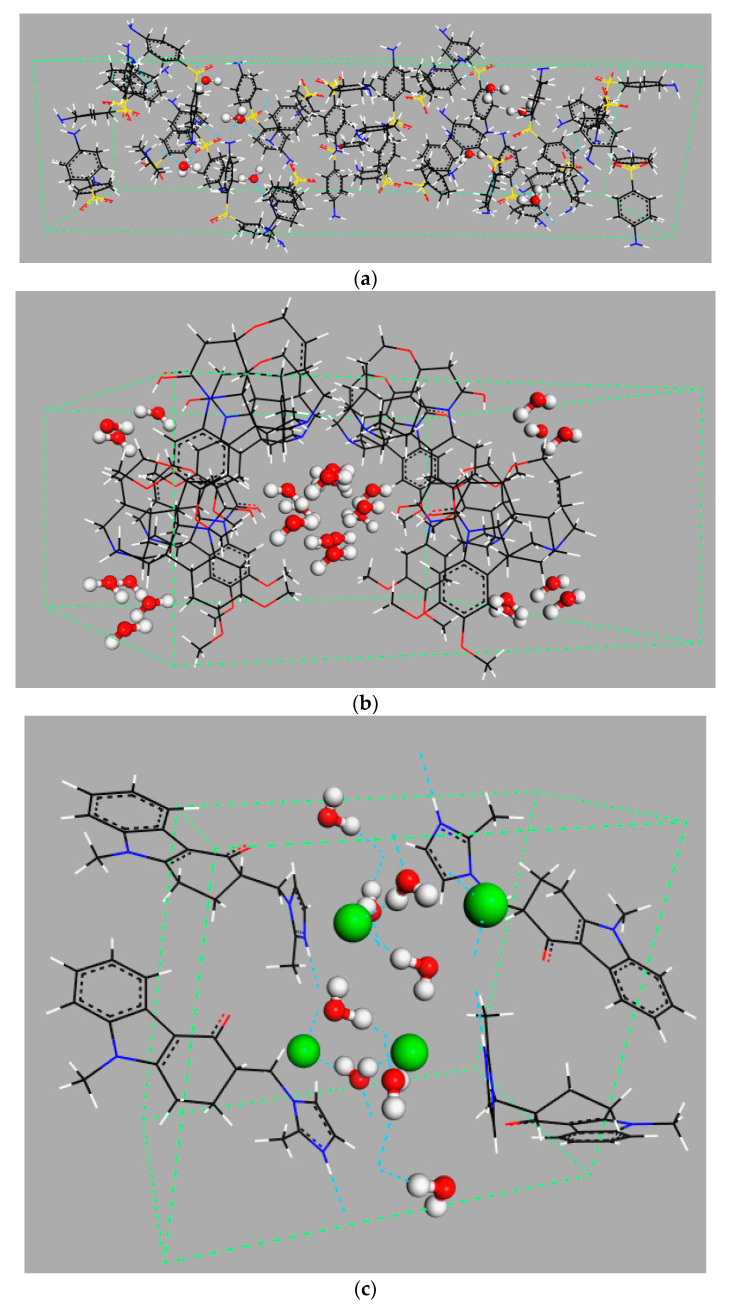
Examples of different types of hydrates: (**a**) isolated hydrate, dapsone; (**b**) channel hydrate, brucine [11]; and (**c**) ion-coordinated hydrate, ondansetron hydrochloride [12]. Crystallographic structures were downloaded from the Cambridge Crystallography Data Centre (CCDC) [13] with the following reference codes: ANSFON (dapsone hydrate), YOYZIX (brucine hydrate), and YILGAB (ondansetron hydrochloride dihydrate). Ondansetron hydrochloride: chlorine is marked in green.

Considering the structure, the most common are layer, void, and channel hydrates (Figure 1). They can be also divided into stoichiometric and non-stoichiometric ones. Stoichiometric hydrates are composed of a constant number of water molecules located in clearly defined structure elements like channels [7]. On the contrary, non-stoichiometric hydrates have a variable number of water molecules incorporated in a crystal lattice and, in this case, their naming, for example, dihydrate, usually indicates the maximum number of H_2_O molecules present in a structure. Non-stoichiometric hydrates can occur as channel or void hydrates, but the solvent molecules are disordered because of the weaker H-bonds with the host molecules, when compared with stoichiometric hydrates [8]. As a result, water diffusion out of these structures is much easier. Furthermore, their hydration level depends highly on the humidity in the surrounding atmosphere. Moreover, non-stoichiometric hydrates are much less prone to collapse after water removal. This phenomenon is characteristic for stoichiometric hydrates, especially when they form large channels or voids. In such cases, distortion of a complex H-bonding network that was stabilizing the stoichiometric hydrate leads to completely new molecular arrangements. Less prevalent among API hydrates are ion coordinated hydrates (known also as ion-associated hydrates) [9] and isolated site hydrates. In case of the latter, water molecules interact solely with the host molecule, but not with each other [10].

Statistically, approximately one-third of pharmaceutical solids exist in at least two forms, differing in the level of hydration [14]. In most cases, in comparison with anhydrous forms, hydrates are thermodynamically more stable under normal conditions. As a result, they are less prone to dissolve in water and, consequently, they usually exhibit lower bioavailability, which is an obvious disadvantage in terms of their therapeutic applications [15]. This key property can be improved by the formation of hydrate co-crystals [16] or application of selected excipients [17,18]. On the other hand, hydrates show better compressibility and tabletability than anhydrates, are less affected by wet granulation process, and are less susceptible for the tablet storage conditions like temperature and relative humidity (RH). In such cases, it could be reasonable to find and produce the most stable API hydrate to prevent the form alteration during production or storage. The choice between a hydrated and anhydrous form of an API is very often a critical factor for solid dosage forms’ stability and their mechanical behavior [19,20,21].

Nevertheless, there are some exceptions from the above given principles. For example, erythromycin dihydrate shows a more efficient dissolution rate than the respective monohydrate and anhydrate [22,23]. Theophylline monohydrate exhibits better solubility in water than its anhydrous form [24]. These cases indicate the variability in properties of hydrated APIs and necessity to perform comparative studies between the differently hydrated forms in each particular case.

While, from the legal point of view in most countries, for each polymorphic form of an API, a separate patent may be assigned, the situation is more complicated for hydrates. More specifically, in some countries, the given two forms of API differing in hydration level can be perceived as legally the same forms, while this is not the case in others. For example, varying in hydration levels, different forms of cefdinir have received individual patent claims in the United States [25]. By contrast, however, the Brazilian and Argentine Patent Law Guidelines exclude the possibility to patent solvates or hydrates, because they both consider them to be discoveries and not new inventions [26,27]. Nonexistence of a worldwide coherent law on this topic has already brought court trials.

Equally disordered is hydrates’ representation in pharmacopoeias, as some of the hydrates are included in separate monographs and some are not, without any clear justification. In pharmacopoeias, one can find only those hydrated forms of drugs that are most commonly used, as well as hydrates of widely applied excipients like lactose monohydrate [28]. For example, in the International Pharmacopeia [29], one can find separate monographs for both caffeine anhydrate and caffeine monohydrate. However, in the case of carbamazepine, a monograph for the anhydrous form only can be found, even though carbamazepine dihydrate is a well-known and commonly applied form of this API [30].

The above shortly enumerated various issues regarding APIs’ hydrates indicate the complexity of the discussed subject, which can also be a real challenge for the pharmaceutical industry. However, when properly employed, the ability of most of the APIs to form solid hydrates can be also a unique opportunity to improve their stability, processability, or biopharmaceutical properties or even to patent a new solid state form, similarly as in the case of polymorphs. However, for these purposes, a detailed analysis of hydrated pharmaceuticals is essential.

This review aims to compare the analytical methods, both instrumental and computational, usually applied in API hydration studies. A particular emphasis is put on their strengths and limitations, supported by the references to the recently published works in which they were successfully applied. The intention of the authors was not to describe in a very detailed way each of the analytical methods, but rather to show their applications and present their capabilities in the analysis of APIs hydrates. As most of the methods described in this work can be used to study both the structure of hydrates and their stability, as well as the hydration and dehydration processes, firstly, selected analytical techniques are introduced and, subsequently, their reported applications are summarized. The aim of this study was, besides presenting the recent advances in this topic, to facilitate the choice of the proper analytical method when exploring the APIs’ hydrates.

## 2. Methods Applied for Analysis of Hydrates

### 2.1. Structure Determination

A starting point in the organic solids analysis is usually detailed determination of their composition and structure. It is especially interesting and important in terms of hydrates, because often, not only polymorphic forms transition, but also change of the hydration degree may occur when the sample is exposed to various temperature, pressure, or humidity conditions.

The difficulty of studies dealing with pharmaceutical hydrates lies in the fact that two different types of molecules are present in a crystal structure, namely API and water. This triggers a stepwise analysis. Firstly, an insight into the number of water molecules per the host substance molecule is needed. This is consistent with contradistinction between stoichiometric and non-stoichiometric hydrates. For this purpose, thermo-analytical method TGA (thermogravimetric analysis) and gravimetric method DVS (dynamic vapour sorption) are usually used. Together with another thermo-analytical method DSC (differential scanning calorimetry), they allow detection of mass gain or loss (due to change of the water content) [31], determination of enantiotropic/monotropic relationship between polymorphs [32], as well as melting or crystallization temperatures [33,34]. Usually, DSC and TGA require a smaller amount of sample (3–10 mg) than DVS (10–30 mg). However, those three methods have some drawbacks and probably the most significant one is the destruction of a sample during the analysis.

It should be also taken into account that these methods not always can be applied for investigation of a mixture of compounds, for example, preformulates or whole drug products in which, besides APIs, excipients are also present. This always depends on a particular example taken into consideration and a type of physicochemical property that aims to be determined.

When a hydration stoichiometry is known, more precise insight into the crystal structure is provided by applying SCXRD (single crystal X ray diffraction), PXRD (powder X ray diffraction), ssNMR (solid state nuclear magnetic resonance spectroscopy), FT-IR (Fourier-transformed infrared spectroscopy), and Raman spectroscopy techniques, as well as computational approaches. They can uniquely identify the crystallinity of API and they are used to determine the exact structure of a hydrated crystal, including the positions and dynamics of water molecules. In the last stage, the analysis of (de)hydration process is sometimes studied. In order to perform it properly, both the knowledge of the structure and results of thermogravimetric approaches are essential.

#### 2.1.1. Structure Determination Techniques

In order to determine the structure of a hydrate, single crystal X-ray diffraction (SCXRD) is usually used. It is the most informative, especially when determining the accurate atoms positions, but at the same time, it is a demanding method. This is because of the usually higher cost of analysis, but more importantly, because of the requirement of a stable crystal of usually a minimum of 0.1 mm in size [35]. Often, this condition cannot be fulfilled, sometimes solely owing to the nature of the sample, as many organic solids do not form stable crystals of the size proper for the SCXRD analysis.

SCXRD is a non-destructive and most willingly used method applied in order to solve a crystal structure. It should be noticed, however, especially in the analysis of hydrates, that the proper determination of the hydrogen atoms’ positions may be very difficult for several reasons. First, the hydrogen atom has only one electron, and thus a very low scattering factor. Further, the electron density distributions from this one electron around hydrogen atoms are usually displaced or pulled towards the bonding regions. Another reason may be the dynamics in the crystal lattice in general and particularly motions involving H atoms that form hydrogen bonds. Finally, the number of water molecules, and thus H atoms forming them, may vary or be significantly disordered in the non-stoichiometric hydrates.

**Figure 2:**
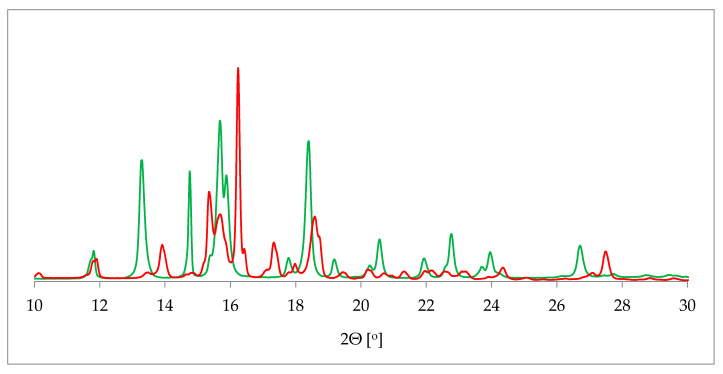
Comparison of powder X-ray diffraction (PXRD) diffractograms of 17-β-estradiol anhydrate (red) received by heating the 17-β-estradiol hemihydrate (green). Source: author’s archive, more details in [38].

One of the most commonly applied methods in the crystal structure studies is powder X-ray diffraction (PXRD). PXRD delivers data on the unit cell parameters, and thus sometimes serves as an alternative route to a single crystal X-ray diffraction (SCXRD) when the latter is not attainable [36]. Further, in contrast to SCXRD, PXRD can be applied to study the mixture of solids and, in some cases, determine the quantitative phase composition [37]. More specifically, in terms of hydrate investigation, PXRD helps to differentiate between the hydrated and dehydrated form of the substance. This is plainly visible as a drastic change of a pattern on the diffractogram [38] (Figure 2).

However, PXRD requires complicated interpretation and demanding refinement in order to provide such detailed information on the crystal structure as SCXRD, especially in terms of the atoms’ positions in the unit cell. Nevertheless, applying synchronically different techniques, with an exclusion of highly demanding SCXRD, it has already been successfully used to determine the structure of new crystalline compounds, including APIs’ hydrates [39,40,41]. What is more, PXRD patterns can be quickly calculated based on the crystal structure files with a high accuracy using specialized software (Reflex) [42]. Such an approach can be used to refine the experimentally obtained data or to differentiate between various polymorphic forms, including hydrates.

**Figure 3:**
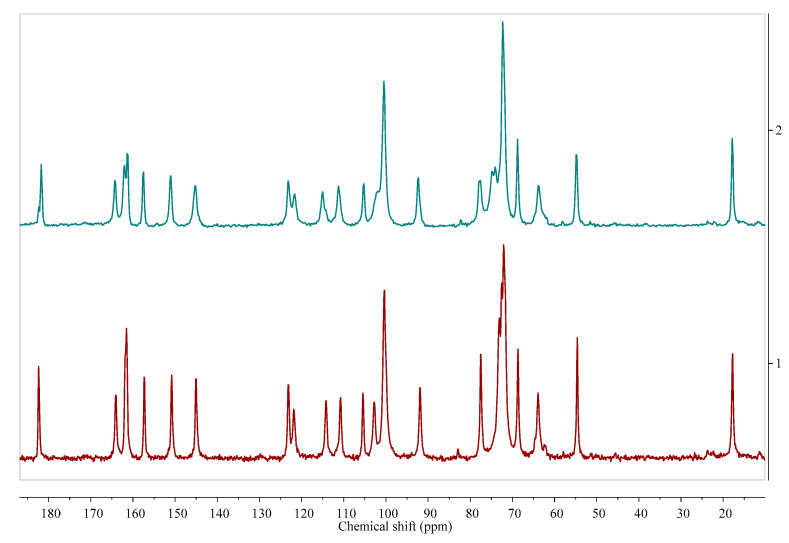
Comparison of ^13^C cross-polarization (CP) magic angle spinning (MAS) solid state nuclear magnetic resonance spectroscopy (ssNMR) spectra of diosmin monohydrate (red, 1) and anhydrous diosmin (blue, 2). Source: author’s archive, more details in [46].

While diffraction-based analytical methods, both PXRD and SCXRD, can be used in the cases of crystalline systems, the next method, solid state NMR, can be used to study both crystalline and amorphous materials (Figure 3).

Solid state NMR (ssNMR) represents a totally different approach than the diffraction methods. It is a non-destructive technique that allows qualitative and in some cases also quantitative measurements. However, ssNMR analysis is relatively expensive and has a long data acquisition time [43]. Intuitively, when applying ssNMR to the study of pharmaceutical hydrates, one could think of registering the ^1^H or ^17^O spectra. However, those kinds of experiments are rarely performed because of the broad overlapping signals in the solid state ^1^H NMR spectra and quadrupolar character of the ^17^O nucleus. A very promising way to overcome the problem of broad signals in the solid state ^1^H NMR spectra is ultra-fast magic angle spinning (MAS). For example, this approach has recently been successfully used to differentiate the anhydrous and monohydrate form of theophylline [44]. The most commonly performed ssNMR analysis experiments are ^13^C and ^15^N, combined with MAS and cross-polarization (CP). In the cases of organic solids, those approaches can be used to distinguish between the forms at different hydration levels (Figure 3), though such experiments do not provide any direct information on water within the crystal structure. Moreover, especially to study the hydration and dehydration processes, ^2^H NMR experiments are being performed using D_2_O in the crystallization process or exposing hygroscopic samples in the contact with the vapor of D_2_O [45]. However, while this approach can be used to investigate the hydrogen bond dynamics in a crystal lattice, it should be noticed that, because of the isotope effect, both the structure and kinetics of hydration/dehydration may be different when D_2_O is used instead of H_2_O.

**Figure 4:**
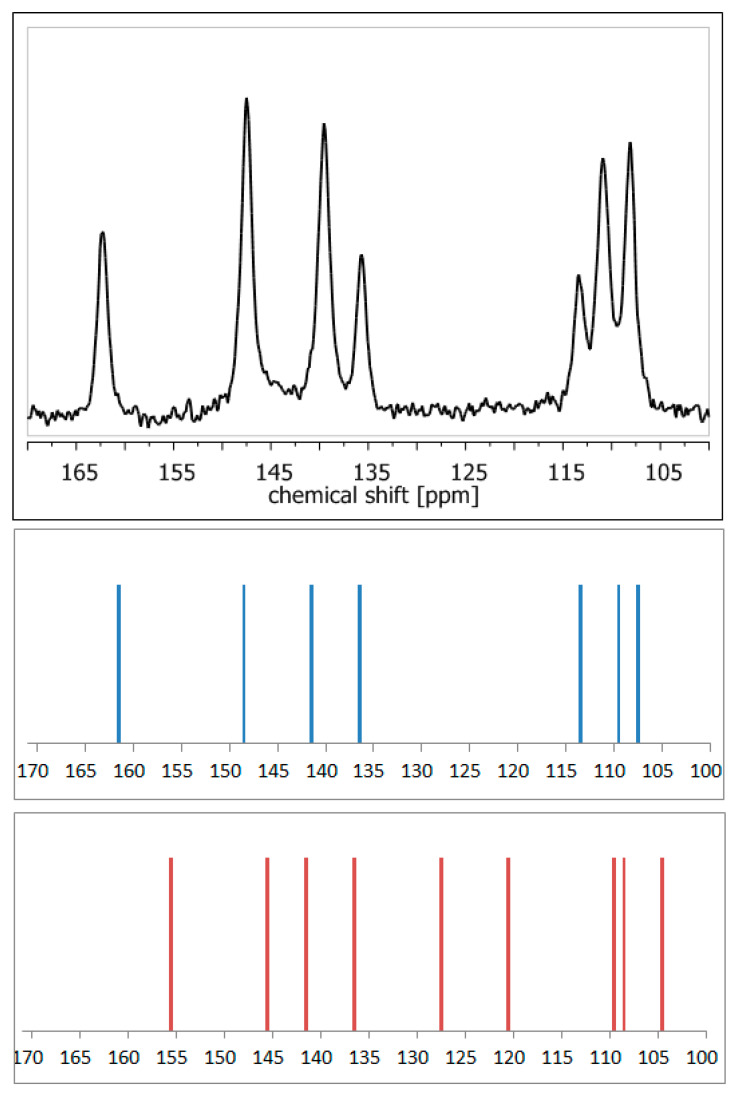
^13^C NMR spectra: experimental of anhydrous elagic acid (black) and simulated using CASTEP GIPAW density functional theory (DFT) calculations results for anhydrous elagic acid (blue) and elagic acid dehydrate (red). Source: author’s archive, more details in [54].

ssNMR measurements are often combined with precise quantum chemistry calculations, which facilitates or even enables proper assignment of NMR signals [47]. Application of DFT (density functional theory)-based GIAO [48] or GIPAW [49] methods makes it possible to theoretically calculate the NMR parameters, such as chemical shielding constants and anisotropy tensors. Thus, it enables validation or refinement of the suggested crystal structure. The high accuracy of such an approach has been already acknowledged multiple times [50,51,52]. For example, calculations of NMR shielding constants in order to simulate the NMR spectra using available crystal structures enable rapid confirmation of whether the analyzed crystal form of a compound is a hydrate or anhydrous one, without the need for any external standards (Figure 4). Nowadays, such a combination of ssNMR and computational methods is so widely used that such an approach has already been defined as ‘NMR crystallography’ [53].

**Figure 5:**
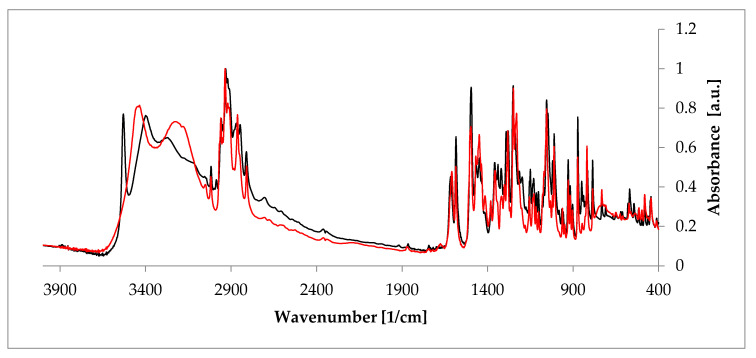
Comparison of the Fourier-transformed infrared spectroscopy (FT-IR) spectra of anhydrous estradiol (black) and estradiol hemihydrate (red). Source: author’s archive, more details in [38].

Apart from PXRD and NMR described above, probably the quickest and easiest methods used to investigate hydrates are FT-IR and Raman spectroscopies, which are complementary to each other. The presence of the unique bands makes it possible to doubtlessly recognize a crystal substance [55]. These two methods are of a special utility in research performed on hydrates because the presence of water can be recognized owing to signals from the hydroxyl group. The representative stretching band of the hydroxyl group appears at a frequency above 3000 cm^-1^ [56]. Another interesting aspect is investigation of a hydrogen bonds’ net created as a result of the presence of -OH groups. Often, a relationship between the peak position of such an H-bond and its strength can be defined. Typically, the higher the wavenumber of the O-H peak (even about 3500 cm^-1^ [57]), the less strong the bond between water and a host molecule in the given hydrate. This, in turn, delivers a suggestion on the overall hydrate structure, because, depending on the character of a host substance, either channel or void hydrate is better stabilized. Consequently, FT-IR and Raman spectroscopies enable analysis of changes in hydrogen bonding that occur during hydration/dehydration process. A good example is rearrangement of this network when estradiol hemihydrate undergoes dehydration (Figure 5) [58]. Moreover, these methods can be applied to identify different polymorphic crystal forms among hydrates. It is based on a careful analysis of the fingerprint spectral region.

However, when solely applying FT-IR or Raman spectroscopy, it is not possible to determinate a completely unknown structure. Moreover, especially in the FT-IR transmission technique, because of the longer preparation process, a risk is posed by the environmental humidity, which could alter the analysis [59]. This is why, for the highly absorbing probes, ATR-IR (attenuated total reflectance) is used as it requires no sample preparation and is also a non-destructive method [60].

A combination of the above mentioned techniques helps to differentiate between various types of hydrates, mainly between channel and isolated site hydrates. In case of the first ones, FT-IR shows sharp OH-bands at relatively low frequencies, while in TGA, rather wide weight loss ranges are observed, and DSC presents broad endothermic peaks. On the contrary, ion isolated hydrates are characterized by sharp dehydration endotherms in DSC and narrow weight loss ranges in TGA [61,62].

A completely different approach to determine the structure of small organic molecules is presented by the new method: cryo-electron microscopy/microcrystal electron diffraction CryoEM/MicroED [63]. It enables collecting the high-quality MicroED data from nanocrystals, which results in atomic resolution (<1 Å) crystal structures. CryoEM/MicroED requires a small amount of sample, very simple preparation, and can be conducted in a few minutes. One of its most important advantages is the fact that it can be applied to derive a crystal structure from seemingly amorphous powders.

#### 2.1.2. Purely Computational Structure Determination Techniques

Crystal structure prediction (CSP) is an already widely accepted multi-step methodology used to computationally determine the structure of a crystalline material [64,65,66,67]. In the field of hydrate studies, it can be used to predict the crystal structure of a hydrate of known stoichiometry. At first, molecular mechanics (MM) is used in order to generate and rank possible compound conformations. Afterwards, selected conformers are subjected to quantum mechanics calculations. The former are performed usually on single molecules, and the latter on whole crystal structures. This enables observation of conformational polymorphism [68] in the first case and packing polymorphism [68] in the second. The (calculations performed at temperature 0 K) lattice energies derived in this process could be qualified if kinetics factors (like temperature) were included. For that purpose, time- and computational power-consuming molecular dynamics (MD) must be applied [69]. Including the entropy input is of a high importance because it means that the zero-point energy (ZPE) will be calculated. It has been proven that the ZPE contributes the most to the free energy differences between hypothetical similar structures [70]. Consequently, its neglection leads to the overestimation of the number of structures generated [64,71]. Furthermore, neglection of the entropy contribution prevents the investigation of the enantiotropically-related polymorphs [72].

Lattice energy is calculated on the basis of the inter- and intra-molecular forces. For example, in the Polymorph Predictor software [73], which is one of the packages used for performing CSP, a two-step procedure is implemented. Firstly, various molecular conformations are generated, and afterwards, optimal hydrogen positions or chemical groups orientations are defined. This process delivers both inter- and intra-molecular energies of the investigated structure, which together form lattice energy.

The target of CSP application in the studies of hydrates is the same as in the case of other molecules; that is, it helps to elucidate the structural information obtained from the experiment, and proposes a variety of structural models that could possibly be the new thermodynamic stable hydrates or polymorphs of the existing hydrates [74].

CSP is usually used when there is no structural information on the investigated molecule available. Its results direct the future experimental approach or support the previous assumptions on the existence and/or structure of the hydrate, as has happened in the case of orotic acid crystal forms [75]. Application of CSP enabled to characterize the structural disorder, and showed stacking faults as well as small variability in packing indices of the anhydrous orotic acid. Another purpose to apply CSP is a situation wherein the experimental attempts to obtain a proper crystal for the SCXRD measurements have failed. In such a case, CSP delivers viable structures that can serve as an important point of reference for further investigations performed with techniques other than SCXRD [76]. A good example is usage of the CSP- and NMR-based approach for analysis of catechin methanol hemisolvate-monohydrate [77]. In this particular study, an important feature of the computational CSP techniques has been utilized. Namely, the possibility to introduce manual changes into the analyzed crystalline systems. This allows to investigate what is not possible regarding hemihydrates in the automatic procedure of calculations [78].

### 2.2. Kinetics of (De)Hydration Process

The description of a (de)hydration process is mostly based on both experimental and calculation methods. The former encompasses spectroscopic and thermogravimetric methods as well as microscopy and other techniques like Karl–Fisher titration (K–F titration) [79]. This titration enables to determine whether water is adsorbed or incorporated in a crystal structure, the latter meaning that the investigated sample forms a hydrate. It is known in three variants: volumetric, coulometric, and oven coulometric K–F titration [80]. In all cases, the used iodine is proportional to the amount of water, which is calculated on the basis of the titrator consumed during the titration process. To perform oven coulometric K–F titration, the sample is pre-weighted and heated. It is the water vapor that is used in the titration [81]. In this last example, thermogravimetric methods are used in order to investigate the thermal stability of a probe and determine the minimal temperature needed for the oven coulometric K–F approach. In general, K–F titration is a very sensitive method. It is found in the pharmacopeial monographs of hydrates, for example, terpin hydrate [82].

If, during the examination of the process with the application of the thermogravimetric methods, big hysteresis in dehydration is observed, it may suggest that the obtained anhydrate is a stable one and it can be analyzed in detail separately by TGA, PXRD, FT-IR, and so on. However, when dehydration is defined as reversible, application of combination of different methods, including Karl–Fischer titration, is recommended [20].

For proper (de)hydration research, an insight into H-bonding structure is needed. Apart from the already mentioned ssNMR and ‘NMR crystallography’, purely computational approaches are also undertaken. For that purpose, DFT calculations involving application of hybrid functionals (most commonly: B3LYP, M05) and polarizable function (an extended basis set like 6-31++G(d,p) or 6-311+G(2d,2p)) are performed [83,84,85,86,87]. Especially, the latter plays a crucial role, as polarizable functions are used to properly define the H-bonding. This helps not only to calculate the energy of the intermolecular interactions (performed also in CSP approach), but also, in further steps, to visualize the H-bonding pattern. Often, it facilitates explanation of the observed (de)hydration mechanism.

In a computational investigation of the (de)hydration kinetics, an important role is also played by calculation of the solvent-accessible volume, which significantly differs for non-stoichiometric and stoichiometric hydrates [88]. Resulting from water loss, possible collapse of a void or conformational disorientation has a direct impact on the above discussed H-bonds network.

### 2.3. Stability Determination in the Industrial Production

The analysis of (de)hydration kinetics is performed to describe the reaction process and to determine whether a transformation between the differently hydrated forms under the given conditions would take place spontaneously, and if so, at what rate [89,90]. The decisive parameters are the energy barrier of water diffusion into the lattice as well as the energy of conformational change [85]. Conversely, the thermodynamic stability investigation is based solely on the reactants of the given process. Described as a function of the Gibbs energy change (Gibbs enthalpy, ΔG) [91], for pharmaceutical purposes, thermodynamic stability is calculated in isothermal-isobaric conditions (NPT ensemble) [92]. Along with molecular dynamics (MD) or compound-water binding energy calculations [93], it can determine the temperature and pressure values under which the global minimum could be achieved. For the pharmaceutical industry, these data are of crucial importance and are equivalent with occurrence of the polymorphic phase transition or desolvation, which could appear during product manufacturing or its storage. These calculations enable to establish a stability order among very often numerous (de)hydrated and amorphous forms of API.

Often, calculations are performed with software based on DFT-D (dispersion correction). Such an approach is already known for high accuracy [94]. However, it must be emphasized that computing the energy difference between hydrate and anhydrate is more challenging than computation of the already relatively well-described polymorph transitions [86,95]. The reason is that, most commonly applied and so far probably most accurate for this purpose, PBE functional overestimates the energy of a transition from hydrate to anhydrate [86,96].

In order to experimentally determine the substance stability, measurement of relative humidity (*RH*) and water activity *a*_w_ is applied. As *a*_w_ informs about the substance solubility [97], its value has a connection with hydrates’ stability order. According to the ICH Guidance for Industry: Q1A(R2) Stability Testing of New Drug Substances and Products, stability measurements for non-sterile products during long-term storage should be performed at specified conditions, which are as follows: 25 °C/60% RH and *a*_w_ < 0.60. This is because *RH* = 60% and *a*_w_ = 0.60 are established as border values for microorganisms’ growth [98]. Regular and accurate testing in the topic is of great importance as only a proper adjustment of RH, *a*_w_ and temperature parameters allows control of the phase formation process and, as a consequence, delivers a stable product [99].

## 3. Hydrates Structural Analysis, Selected Cases within the Last 10 Years

Observed in the last 10 years, rapid development of both experimental and computational methods used in structural analysis of solid state substances has its reflection in a number of recently determined structures of hydrated APIs [58,100,101,102,103,104,105,106]. In the majority of cases, numerous different experimental methods (briefly described in Section 2.2) are used within one study in order to properly determine the structure of a substance [41,77,89,107,108,109]. A separate group of hydrates form hydrated co-crystals, which are quite common among obtained forms of APIs. Despite being more complex than simple hydrates, their analysis unfolds with application of the same methods [62,110,111,112,113,114,115].

In case of APIs’ hydrates, the most commonly applied theoretical approaches encompass CSP [86,88,116,117] and NMR crystallography methods [83,118,119,120,121,122], not both of them at once [123,124]. Because of the common simultaneous application of various analytical methods by the researchers, it is impossible to review the last decade advances in this field method by method. However, it is possible to divide the published studies with reference to the investigated subjects such as structure determination, mechanism of (de)hydratation, stability, and so on.

Determination of a hydrate type is crucial knowledge in order to properly understand the upcoming polymorph or solvate change. In the CSP approach, firstly, water-free high-energy structures are determined with the aim of calculating the solvent-accessible volume [86]. This helps to direct further crystal structure prediction for either non-stoichiometric or other well-structured hydrate forms. For example, for a very similar purpose, water interactions with a host molecule in norfloxacin salts have been put under close investigation [125]. It has been revealed that some of the structures are ion-associated hydrates, while the others belong to typical channel hydrates. This structural difference has a direct impact on the hydrates’ stability as well as their dehydration temperature. Therefore, it delivers the molecular level explanation for the observed results of thermal studies.

Not only kinetic parameters, but also resulting products of the dehydration reactions are influenced by the hydrate’s type. An example is 5-HT2a and H1 inverse agonist [126], for which two zwitterionic hydrates (HyA, Hy2) and three anhydrates (Form I, II, III) are known. HyA is a non-stoichiometric hydrate and has voids clearly separated from each other. On the contrary, Hy2 is a stoichiometric dihydrate with water channels running in a zig-zag manner along the crystallographic b-axis. The consequences of such structural differences are distinct dehydration mechanisms leading to different reaction products. Removal of water molecules from HyA results in anhydrate Form I and II, whereas Hy2 dehydration ends with Form III as a product, and it is the only known way to obtain Form III. These types of investigations are of a crucial importance for the pharmaceutical industry.

An in-depth investigation of dapsone hydrates [116] is a good example of a work that involves a variety of methods and has been performed recently. Application of PXRD allowed to describe H-bonds motifs, while CSP revealed different structural arrangements among forms I, IV, and V. Moreover, new hydrate V was found and an unusual stoichiometry of 0.33-Hy and its isostructural relationship with Hy_dehy_ was described. What is more, for 0.33-Hy, it has been revealed that water molecules are located at the isolated sites of a dapsone molecule framework, instead of being situated in channels [127], as usually happens for such non-stoichiometric hydrates. The amount of gathered data enabled construction of a very useful (de)hydrogenation chart.

During hydrate examination, a specific accent must be put on the H-bonding investigation. This is because these are the H-bonds which are one of the main reasons for the structural alteration of a non-hydrate [128,129], as they are formed not only between water and API molecules, but also in the form of water bridges [130]. All these aspects contribute to the formation of complex H-bond networks. In the case of etoricoxib, a thorough analysis of a newly found hydrate led to a hypothesis that the reason for a hydrate existence could be a lack of the H-bonding donor groups in the starting structure [131]. It was only a water molecule that compromised this deficiency and, in the given conditions, enabled creating a stable etoricoxib form. An analogical example is the case of morphinans for which hydrates are preferable to anhydrates owing to the presence of H-bonding donors/acceptors, which enables creating a more stable H-bonding network [132].

What is more, H-bonding is also one of the underlying elements for both conformational and packing polymorphism, also observed among hydrates. The studies targeted on the latter reveal whether mono- or poli-layered systems are formed [87,133] and deliver data on the arrangement of molecules within a crystal unit. For example, a vibrational and thermogravimetric analysis of codeine phosphate sesquihydrate [134] led to a conclusion that, depending on the solvation method, different hydrate polymorphs are obtained and their molecular arrangements are often different from the ones present in the commercial APIs.

## 4. Mechanism of (De)Hydration, Selected Cases within the Last 10 Years

**Figure 6:**
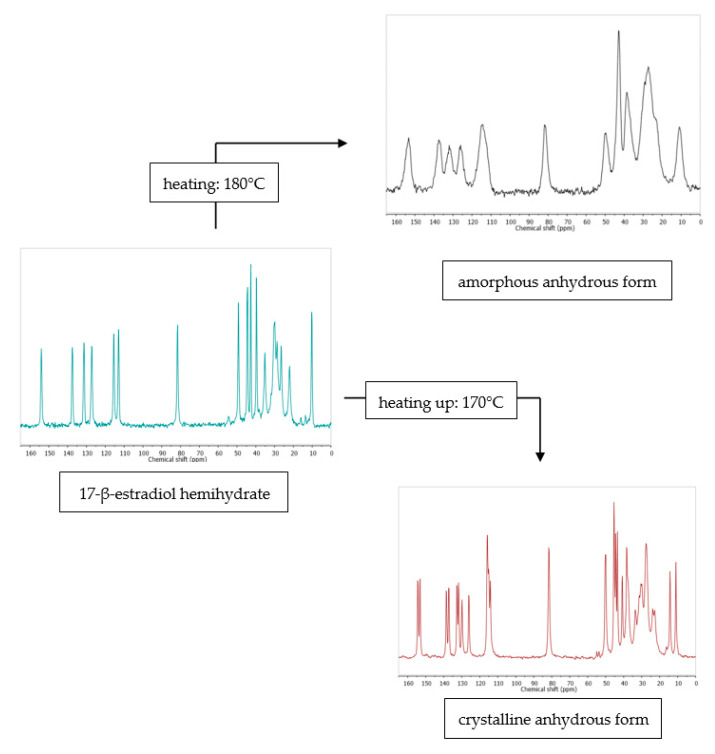
^13^C ssNMR as a method that can be used to study the effect of dehydration temperature on the received anhydrous product form. More information in [38]. Source: author’s archive.

The mechanism of (de)hydration is obviously strongly related to the substance’s structure. Raised in the last section, the topic of hydrate channels is a very important issue. A good example is the structure of mildronate dihydrate [135]. Its dehydration process proceeds in a one-step manner, without an intermediate monohydrate, as in most other dehydration cases. It is a consequence of a presence of very large channels, which triggers a loss of whole water at once, instead of proceeding in stages. This example plainly shows how structural aspects are linked with the dehydration kinetics and explains why kinetics of water escape, H-bonding network breakdown, and molecules’ rearrangement within a crystal are analyzed simultaneously [84]. It has been shown even more clearly in the example of fluconazole [85]. Both structural and kinetic aspects make a hydrated fluconazole form preferable. These are the hydrate structural similarity to anhydrate and low hydration barrier. Unfortunately, the formation of a hydrate lowers drug’s solubility and, as a result, reduces its bioavailability. Such an example shows that the production of pharmaceuticals prone to hydration forms a real challenge for the industry. It also indicates that only complex structure-kinetics investigations allow for proper understanding of the dehydration process and its implications (Figure 6).

The available methods enable a step by step [136] observation of the changes occurring during dehydration or the water uptake [137]. These studies deliver information that is very important for the pharmaceutical industry. Namely, they can reveal the existence or non-existence of an intermediate in the dehydration process [138], as well as define the kinetics of a conversion of an amorphous substance into a crystalline upon the dissolution [139]. A perfect example of the strong influence of hydration conditions on the received hydrate form is the case of sodium naproxen [140]. An anhydrous form changes in a one-step process into a dihydrate I, but when the hydration is conducted in two steps, dihydrate II emerges. Many more far reaching structural consequences can cause dehydration in a hydrate co-crystal. Water loss from carbamazepine-nicotinamide cocrystal hydrate induced new co-crystallization between anhydrous carbamazepine and nicotinamide [141], ending up in a completely new structure. All the changes have been observed thanks to step by step PXRD analysis.

Another crucial aspect for the industry is spontaneous water absorption. In such a process, anhydrate carbamazepine converts into the hydrate form I, whose different crystallinity leads to a significant decline of the drug’s bioavailability [142]. An adverse process is of no less importance as some APIs undergo water desorption-induced phase transition. Correlation of a weight loss with RH requires application of, for example, water vapour sorption measurements and Raman spectra, to obtain diagrams of sorption–desorption cycle. This gravimetric-spectroscopy methodology has already been proposed and effectively applied to determine the optimal storage conditions of drugs [90].

A related topic that is of a huge industrial importance is reversibility of the hydration. Such a situation concerns anhydrate and trihydrate of alendronate monohydrate [116]. Thermogravimetric measurements together with computational methods deliver a possible molecular-based explanation for this phenomenon. The suggested reason is the fact of firstly releasing two non-bonded to the sodium cation water molecules, and only afterwards proceeding with liberation of the third, cation-solvated water molecule. This plainly shows that solvation of cation is stronger than the structural interaction of water molecules with the host molecule. Another example described in detail, which is associated with the hydration reversibility topic, is the case of paroxetine hydrochloride hydrates [143]. Here, on the contrary, it has been proven that no sorption–desorption hysteresis can be depicted due to water presence in channels and not in pores of the hydrates. This was part of a renewed description of paroxetine hydrochloride hydrate form II, which has previously been determined as an anhydrate. However, new research, including PXRD measurements performed at variable humidity, has documented the hypothesis that paroxetine hydrochloride hydrate form II is a non-stoichiometric hydrate. This particular example points out the difficulty connected with differentiation between various inner packing systems of water molecules in hydrates, which represents a challenge for both science and industry. An interesting case is the relation between one of the quinaldine hydrochloride anhydrate (form B) and substance’s dihydrate [87]. Characteristic of stoichiometric hydrates, hysteresis between sorption and desorption isotherms is observed. Nonetheless, it is very small, which indicates high structural closeness of hydrate and anhydrate as well as the fact that this reversible phase transformation has a low energy barrier. This agrees with other experiments indicating that dihydrate is the most kinetically stable form of quinaldine hydrochloride. Once more, such data play an important role in pharmaceuticals’ manufacturing and, furthermore, in their storage.

An important aspect of the (de)hydration kinetics is the number of the reaction steps. It raises the topic of possible metastable intermediate phases. There are a fair amount of examples. At 95% RH and 25 °C, acyclovir anhydrate form II transforms firstly into hydrate 3:2 (form V) and afterwards, under the same conditions, into dihydrate (form VI) [120]. Another example is ondansetron hydrochloride dihydrate. After significant structural rearrangement is that it changes in the first step into hemihydrate and later on into anhydrate [144]. In the first step, the hydrogen bonding is altered. The second stage implies structural differences as well. This time, a void is formed and the volume of the hydrophobic layer is changed. In contrast, a two-step and quite atypical (at first, loss of 1–1.5 water molecules, and later, loss of 2.0–2.5 water molecules) dehydration of catechin 4.5-hydrate triggers no structural changes [145]. The reactions are reversible. For the most precise determination of this very specific process, both experimental (^13^C CPMAS NMR, ^1^H NMR) and calculation (GIPAW-NMR, CSP) methods have been applied.

Another factor that influences the dehydration mechanism is the material preparation process. Non-micronized potential dyslipidemia reducing agent, non-stoichiometric dihydrate, follows a two-step reaction, whereas its micronized form undergoes a one-step dehydration [124]. The two processes differ distinctly. For their description, a combination of TGA, DSC, PXRD, SXRD, ^15^N ssNMR, and DFT has been applied. All the above mentioned examples show how diverse multi-step reactions of (de)hydration are and, at the same time, indicate the need to employ a variety of research techniques.

## 5. Hydrates’ Stability, Selected Cases within the Last 10 Years

Kinetic stability could be briefly defined as a reaction’s probability to occur in the given conditions. However, a kinetically stable compound is often at the same time thermodynamically metastable as it has not reached a global energy minimum because of the high energy barrier of a phase transition [81]. Nevertheless, application of different external conditions could influence the direction of the hydrate-anhydrate reaction. The practical importance of this subject explains numerous scientific articles published on the topic.

The stability order of hydrates/anhydrates is a function of *RH*, temperature, and *a*_w_. [87,116,132,146]. To name an example, gabapentin anhydrate is more stable at lower water fractions and rising temperatures when compared with the drug’s hydrate, whereas gabapentin monohydrate is favorable at higher water fractions and lower temperatures [129]. Such behaviour concerning anhydrate being more stable at lower *RH* and a hydrate at higher *RH* is a general rule. However, at very high *RH*, drug substances are prone to degradation and this is the reason the ICH guidelines suggest performing stability studies in accelerated conditions of 40 °C/75% RH [147]. Though, there are some exceptions, like levothyroxine sodium pentahydrate which remains stable even at high *RH* rates and is unstable at lower ones [148]. The explanation is most probably the presence of unusually high number of water molecules within this hydrate.

Kinetic stability is also an aspect that attracts attention while investigating hydrate co-crystals because it is connected with solubility, which in turn determines bioavailability [149,150,151,152,153]. One of the experiments showed that kinetically stable imatinib hydrate, after associating syringic acid, forms a metastable co-crystal with higher permeability through membranes [154]. Similar results deliver examination of griseofulvin-acesulfame hydrate. It presents remarkable thermal stability at different RH up to 13 weeks and has an improved dissolution rate when compared with the griseofulvin hydrate [155].

Stability is a decisive factor for storage aspects [156,157]. It depends on the material’s purity [86,158]. An addition of other phase can induce nucleation of another form and, as a consequence, a polymorphic transformation could take place [158]. Later on, it could influence the process of formulation preparation as well. One of the first stages of drug industrial production is grinding, which can induce polymorphic changes and (de)hydration [159]. For this reason, studies including this process are performed. For example, neomycin sulphate hydrate lost its water after 2.5 h of grinding, but a hydrate could be restored after storage in 71% and 99% *RH* [157]. Conversely, theophylline and caffeine anhydrates transformed into hydrates after 5 min long grinding with water [160].

The next step in the drug industrial production is often wet granulation. As the storage stage, it represents a potential risk of unwanted hydration. For example, in the case of theophylline, wet granulation shows a very specific impact on the tableting process; that is, at 40 °C, theophylline anhydrate is subjected to the following transitions: THA→THM→THA, where THA is theophylline anhydrate and THM is theophylline monohydrate. However, when wet granulation is performed at 60 °C, the compound stays in a form of THA the whole time [161]. Irrespective of the fact that, in the end, in both cases, THA is maintained, the differences in tablet hardness, disintegration time, and dissolution rate, assigned to the hydration changes during granulation, are detectable [161].

## 6. Influence of Excipients on API Hydrates

Excipients can influence APIs’ nucleation or their solubility [108]. Investigation of the whole formulation instead of merely the active ingredient poses a challenge in the pharmaceutical analysis for each type of API. In API hydrates, it is probably even more pronounced owing to the presence of water in APIs’ crystal lattice [77,119,123]. The majority of cases reported on the excipients’ role in a drug formulation where API has a form of a hydrate concern polymers. Their direct influence on the (de)hydration process has been analyzed in detail. For instance, a study on sodium naproxen hydrate indicates that, in tablet pressing conditions, polivinylpyrrolidone (PVP) delays, whereas microcrystalline cellulose (MC) facilitates, transformation into the anhydrate [137].

However, because, in most of cases, polymers prevent an opposite reaction, namely hydration, most studies concentrate on this aspect while examining formulations that include hydrated API. The polymers’ effect depends on both the manufacturing process parameters and polymers’ type. For example, inhibition of the caffeine hydrate formation by PAA (polyacrylic acid) is dependent on pH values. The highest process intensity is observed at low pH values. Moreover, the same study shows that PVA (polyvinyl alcohol) with a higher molecular weight inhibits caffeine dehydration more than PVA with shorter chains [109]. Similar results are delivered in the study on olanzapine. PVP K30, even if added at a very low concentration (0.002%) to the formulation during the wet granulation process, effectively prevents formation of olanzapine hydrate [162]. Another study indicates that potent inhibitors of olanzapine hydrate-formation are PVP and HPC (hydroxypropyl cellulose) [163,164]. Interestingly, PEG (polyethylene glycol) stimulates a further process up to higher hydrates. Another example is the case of carbamazepine, whose hydration was inhibited by short chain substituted cellulose like HPMC (hydroxypropylmethyl cellulose) and MC. The same trend has been observed for PVA and PVP; that is, longer chains exhibited lower efficiency [155].

Water originating from excipients can alter the solubility rate of API, and thus influence the drug’s bioavailability [62]. Especially excipients with high water-absorbing potential, like MC or HPC, can delay the start of a hydrate formation in an aqueous environment (e.g., wet granulation), as has been reported for piroxicam [116]. Nevertheless, sometimes, excipients also cause a stability decrease in formulations containing API hydrates [128].

A wide range of excipients’ impact on the final drug product justifies the importance of research on this topic. The complexity of the problem requires application of different analytical techniques. To determine the structural purity, Near Infrared (NIR) is used because, unlike the thermal methods, this technique may overcome interference from excipients [120]. To observe anhydrate–hydrate changes, mostly Raman spectroscopy is applied [62,109,161], whereas explanation of excipients’ protective effect on anhydrate APIs is provided by PXRD and NMR measurements [55,77]. This hydration prevention is determined by dense H-bonding network emerging between API and polymers and by the hydrophobic layer formed by excipients around the active ingredient. These factors prevent water molecules from coming into contact with the host substance and, as a result, no hydrate in drug formulation is observed.

## 7. Analysis of Commercial Solid Dosage Forms

**Figure 7:**
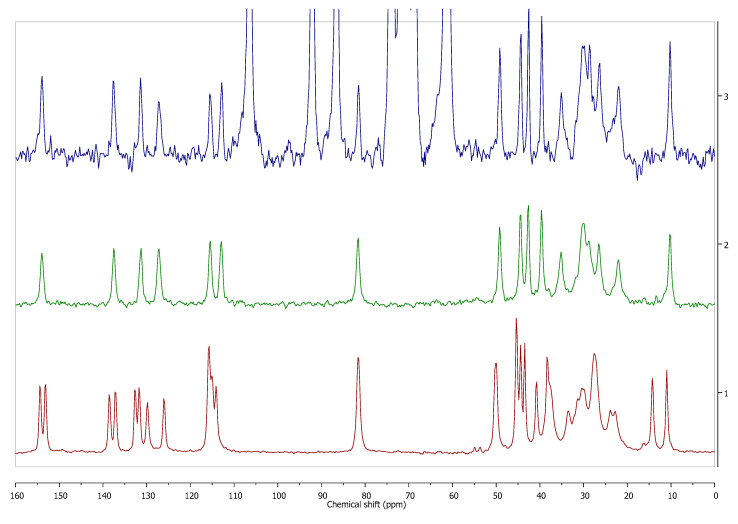
Estrofem mite ^®^ (3), 17-β-estradiol hemihydrate (2), and anhydrous 17-β-estradiol (1) ^13^C CP MAS NMR spectra. Solid state NMR analysis enabled to determine which form of 17-β-estradiol is present in the commercial dosage form, despite the low content of active pharmaceutical ingredient (API) (<5%). Source: author’s archive, more details in [38].

Solid dosage forms are the most popular methods of drug delivery due to their numerous advantages like convenience of administration and facility of mass production. However, solid state pharmaceutical analysis, suffers from some inconveniences. For example, the inability to separate the components of the homogenous samples such as solid drug formulations and the low method sensitivity when compared with the solution methods. However, only the solid state analysis can provide information on the solvated forms of APIs used in the formulation. Though all of the analytical methods described in the first section of this review (PXRD, FT-IR, Raman, ssNMR, and DSC), with the exception of SCXRD, can possibly be used in the analysis of solid dosage form, and each of them have their advantages and disadvantages. When choosing the appropriate one in a particular case, the attention should be paid to the qualitative and quantitative composition of the excipients in the solid dosage form, which may interrupt the analysis, as well as the API content. In a recent study [38], the performance of selected solid-state analytical methods (PXRD, FT-IR, and ssNMR) in the analysis of the solid drug forms with low concentration of an active ingredient has been compared. The authors found that the only method that unambiguously confirmed the presence of a particular solvated form of API in the studied drug was ssNMR (Figure 7). Because of the possibility of modification of pulse sequence and the manipulation in the ssNMR experimental registration parameters like recycle delay, inversion recovery CP evolution time, and CP contact time, it was possible to observe the signals of the chosen components of the drug formulation. The other studied methods (PXRD, FT-IR) have failed because of the overlapping of the excipients’ signals with the signals of an API.

## 8. Conclusions

Polymorphism is a phenomenon of key importance for the pharmaceutical industry. Likewise, the possibility of solid APIs to exist at different levels of hydration represents another way to improve the APIs’ properties by altering their solid state structures. However, differences in the level of hydration can affect APIs’ physical and chemical properties, such as solubility, dissolution rate, melting point, stability, and tabletability. For this reason, a thorough screening for differently hydrated forms of APIs, as well as determination of their structures together with the mechanism and kinetics of (de)hydration, are necessary in the modern drug production process.

Among the instrumental and computational techniques presented in this review, there is none that, used alone, would answer all the questions regarding the above-mentioned structural issues. All of the described analytical methods are complementary to each other. Only when used as a set, they allow precise determination of APIs’ structure and stability. Moreover, in this way, they provide a tool to track structural changes that occur during the production process as a result of different humidity, temperature, or pressure conditions.
